# Integrated bioinformatics and molecular docking analysis reveal potential hub genes and targeted therapeutics in sepsis-associated acute lung injury

**DOI:** 10.3389/fimmu.2025.1684774

**Published:** 2025-10-10

**Authors:** Qiongyan Chen, Yifeng Mao, Shangwen Cai, Xijiang Zhang, Chenghao Zeng, Qingqing Chen, Cheng Zheng

**Affiliations:** ^1^ Department of Respiratory and Critical Care Medicine, Wenzhou Hospital of Integrated Traditional Chinese and Western Medicine, Wenzhou, China; ^2^ Department of Critical Care Medicine, Taizhou Municipal Hospital (Taizhou University Affiliated Municipal Hospital), School of Medicine, Taizhou University, Taizhou, China; ^3^ Department of Gastroenterology, The Second Affiliated Hospital of Zhejiang University School of Medicine, Hangzhou, China; ^4^ Institute of Gastroenterology, Zhejiang University, Hangzhou, China; ^5^ Department of Anesthesiology, Second Affiliated Hospital of Zhejiang University School of Medicine, Hangzhou, China; ^6^ Rehabilitation Center, Taizhou Hospital of Zhejiang Province Affiliated to Wenzhou Medical University, Taizhou, China; ^7^ Neurorehabilitation Center, Taizhou Rehabilitation Hospital, Taizhou, China; ^8^ Taizhou Enze Medical Center (Group), Taizhou Rehabilitation Hospital, Taizhou, China

**Keywords:** sepsis-associated acute lung injury (SA-ALI), hub genes, machine learning, molecular docking, small-molecule drugs

## Abstract

**Background:**

Sepsis-associated acute lung injury (SA-ALI) is a severe complication of sepsis with high mortality. This study aimed to identify key diagnostic genes and potential therapeutic drugs for SA-ALI.

**Methods:**

Transcriptomic data from GSE10474 and GSE32707 were integrated for differential expression and WGCNA analysis. Hub genes were screened using PPI network construction and three machine learning algorithms, and validated by Western blot. Functional enrichment, immune infiltration, and drug prediction (DSigDB) were performed, followed by molecular docking.

**Results:**

Six hub genes (*PGM3, GDF15, GART, GFOD2, E2F2, ATP1B2*) were identified and validated with elevated expression in SA-ALI. These genes were enriched in inflammation, immune regulation, oxidative stress, and tissue remodeling pathways, and showed significant correlations with specific immune cell subsets. Five candidate small molecules were predicted; molecular docking revealed Celastrol had the strongest binding to all six proteins, particularly *GDF15* (-9.988 kcal/mol), while Thiostrepton showed strong binding to *PGM3*, *GFOD2*, and *GDF15*.

**Conclusion:**

Six diagnostic hub genes and two priority candidate drugs, Celastrol and Thiostrepton, were identified for SA-ALI, providing potential biomarkers and therapeutic targets.

## 1 Introduction

Sepsis is a common disease in the field of critical care medicine (1, 2). Sepsis impacts at least 30 million individuals worldwide annually, with a mortality rate reaching up to 20%, according to a World Health Organization report (3). When sepsis progresses to sepsis-associated acute lung injury (SA-ALI), the mortality rate can reach 34% to 45% (4, 5), posing a serious threat to patients’ life and health. The known causes of SA-ALI include various biochemical injuries, severe trauma and post-traumatic infection, shock, and poisoning (6), as well as various infectious diseases targeting the lungs, especially the COVID-19 pandemic that erupted at the end of 2019. Some patients can rapidly progress to SA-ALI, experience respiratory failure, and even death (7). Due to the lack of early diagnostic methods, only about 50% of SA-ALI cases can be identified by clinicians (8). Therefore, the study of early identification of SA-ALI, as well as the research on biomarkers for reducing its mortality rate and improving prognosis, is extremely important.

In recent years, basic and clinical research on biomarkers has not only deepened our understanding of the pathophysiological mechanisms of SA-ALI but has also provided a wealth of biological information, offering greater value for the prediction, diagnosis, and prognosis of the disease (9). Currently, research on SA-ALI biomarkers mainly involves non-protein indicators such as endothelial progenitor cells and exhaled breath condensate (10, 11). Despite some progress in biomarker studies over the past two decades, their clinical application value remains low. The reasons for this may include: (1) an overreliance on single indicators for early warning, diagnosis, or prediction of SA-ALI, which have limited diagnostic value, whereas a combined biomarker approach may improve accuracy; (2) low tissue and disease specificity of some markers, making them easily influenced by other factors; (3) limited understanding of the involvement of certain biomarkers in SA-ALI development; and (4) lack of validation in large-sample clinical studies (12). As a result, there is a pressing need to identify novel biomarkers for the early detection of SA-ALI.

To address these challenges, this study applied bioinformatics analysis to uncover key genes potentially involved in the pathogenesis of SA-ALI and used molecular docking to screen candidate therapeutic compounds targeting these genes. Unlike previous work that has primarily focused on isolated markers, our approach integrates computational biology and drug–target prediction, aiming not only to reveal novel biomarkers for early detection but also to provide innovative strategies for precision diagnosis and targeted therapy in SA-ALI. By bridging the gap between mechanistic insights and translational application, this study offers a new framework to improve the early identification of high-risk patients and to guide the development of effective therapeutic interventions.

## 2 Methods

### 2.1 Original data

**Figure 1:**
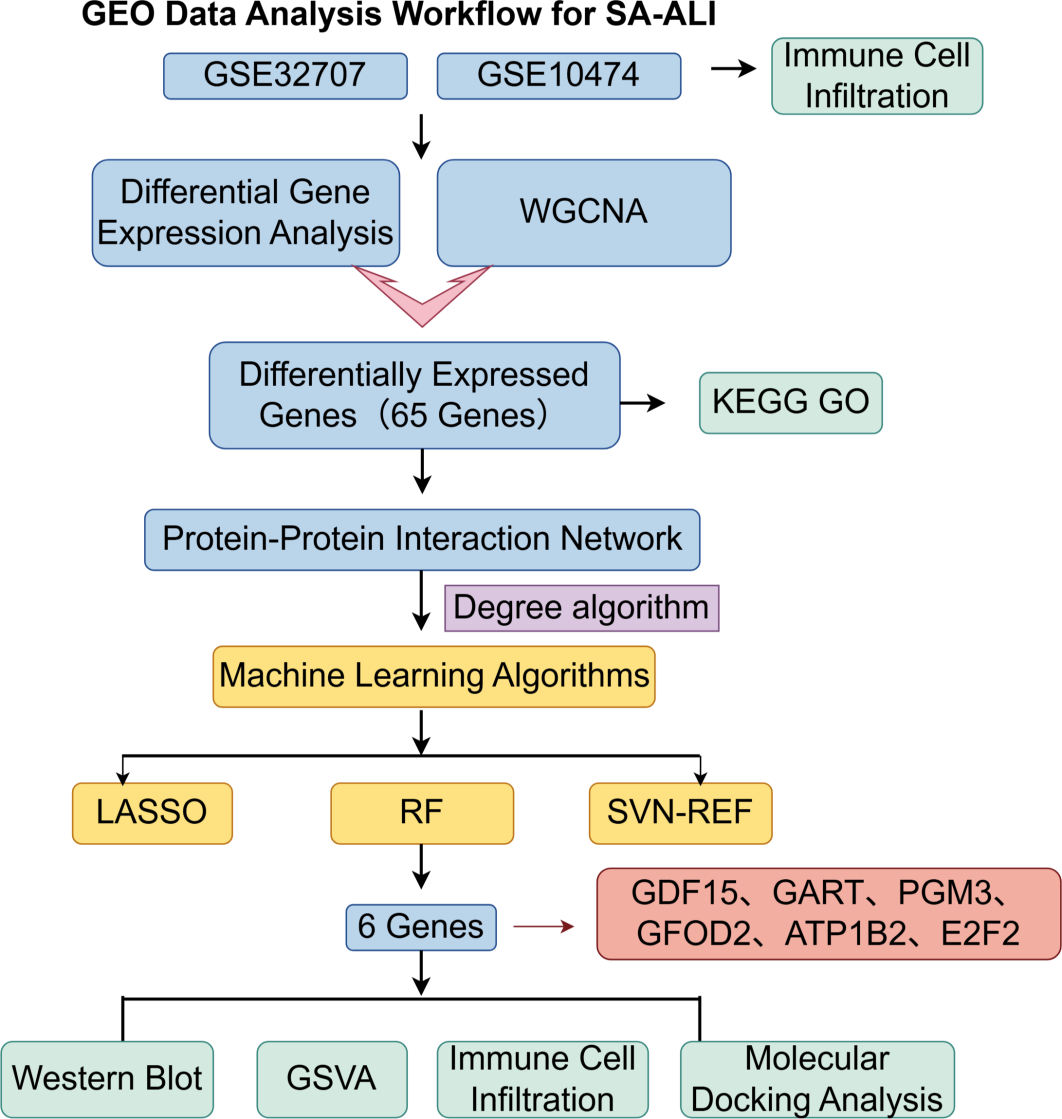
The working flow chart of this study.

In this research, we utilized the microarray datasets GSE10474 and GSE32707, accessible from the Gene Expression Omnibus (GEO) repository (www.ncbi.nlm.nih.gov/geo/). GSE10474, built on the GPL571 platform, comprises 13 blood samples from individuals with ALI and 21 samples from patients diagnosed solely with sepsis. GSE32707, based on the GPL10558 platform, contains 144 blood samples, from which we selected 58 cases of isolated sepsis and 31 cases of SA-ALI for additional analysis. The brief research procedure is illustrated in **Figure 1**.

### 2.2 Differential gene expression analysis

Raw microarray data were log2-transformed and quantile-normalized using the preprocessCore package in R (v4.3.3). Probe IDs were mapped to gene symbols based on platform annotation files; probes mapping to multiple genes were removed, and for genes with multiple probes, the mean expression value was calculated. Batch effects were corrected using the removeBatchEffect function in the “limma” package. For cross-platform analyses, only common genes were retained, and datasets were treated as separate batches before batch correction. Principal component analysis (PCA) plots were generated to evaluate sample distribution before and after correction.

### 2.3 WGCNA network construction and module identification

Co-expression networks were constructed using the Weighted Gene Co-expression Network Analysis (WGCNA) package in R. Samples were first hierarchically clustered to detect and remove outliers. The soft-thresholding power (β) was determined using the pickSoftThreshold function to ensure a scale-free topology. A topological overlap matrix (TOM) was calculated to assess gene interconnectedness, and modules were identified via dynamic tree cutting. Module–trait relationships were evaluated by correlating module eigengenes with clinical traits. Genes from key modules were selected for subsequent analyses, and the eigengene network was visualized to explore inter-module relationships.

### 2.4 Identification of common DEGs

To identify Differentially Expressed Genes (DEGs), we applied the Linear Models for Microarray (LIMMA) package in R (version 4.3.3) to carry out a comparative analysis between the sepsis-only group and the sepsis-induced ALI group. DEGs were identified using a strict threshold of p-values less than 0.05. Additionally, genes from the WGCNA modules that exhibited the strongest negative correlation with sepsis-induced ALI were also selected. By intersecting the two sets of genes, we were able to pinpoint the DEGs that were common to both, thus satisfying the defined selection criteria.

### 2.5 Enrichment analysis of common DEGs

To further explore the biological functions of the identified shared DEGs, we utilized the Gene Ontology (GO) and Kyoto Encyclopedia of Genes and Genomes (KEGG) pathway enrichment analyses. This was conducted using the “clusterProfiler” package in combination with the “org.Hs.eg.db” database for annotating genes. The species of reference for the analysis was Homo sapiens, and enrichment significance was defined as having an adjusted p-value of less than 0.05.

### 2.6 Construction of protein-protein interaction network

The STRING database (https://string-db.org/) was used to create the PPI network, applying a confidence score threshold of at least 0.4. Cytoscape v3.7.2, along with the CytoHubba plugin (version 0.1), was used to visualize and analyze the network. Hub genes were determined using the Degree algorithm, and the ten genes with the highest rankings were selected for further examination.

### 2.7 Feature selection using three established machine learning algorithms

Candidate hub genes were refined using three machine learning algorithms—Least Absolute Shrinkage and Selection Operator (LASSO), Support Vector Machine–Recursive Feature Elimination (SVM-RFE), and Random Forest (RF)—with a fixed random seed of 123 to ensure reproducibility. LASSO regression was performed using the “glmnet” package in R with 10-fold cross-validation to select the optimal lambda value (“lambda.1min”), while SVM-RFE was conducted with the “e1071” and “MSVM-RFE” packages to identify the subset with the highest classification accuracy. RF analysis was implemented via the “randomForest” package with 500 trees, ranking genes by importance and applying a significance threshold of 0.9. The final hub genes were obtained by intersecting the results from all three algorithms.

### 2.8 Clinical sample collection

This study was conducted in accordance with the Declaration of Helsinki and approved by the Ethics Committee of Wenzhou Hospital of Integrated Traditional Chinese and Western Medicine (No. 2024-L076) and the Ethics Committee of Taizhou Municipal Hospital, School of Medicine, Taizhou University (LWYJ2025276). Peripheral blood samples were collected from three patients meeting the diagnostic criteria for SA-ALI and one healthy volunteer confirmed by the hospital’s health examination center as a control. Inclusion criteria were age ≥18 years, provision of informed consent, and, for the study group, fulfillment of ALI diagnostic criteria; exclusion criteria included age <18 years, incomplete clinical data, death during hospitalization or transfer, presence of organic heart disease (e.g., ischemic cardiomyopathy, congenital heart disease, myocarditis), end-stage malignancy, psychiatric disorders or cognitive impairment, or refusal to participate. All samples were centrifuged at 2,000 rpm for 10 min at 4 °C, and the resulting supernatants were immediately stored at −80 °C for subsequent analysis.

### 2.9 Validation of hub gene expression by western blot

To validate the expression levels of hub genes in sepsis-associated acute SA-ALI, WB analysis was performed on serum samples to detect the corresponding protein levels. Total protein was extracted using RIPA lysis buffer containing PMSF (G2008, Servicebio, China) and quantified with a BCA protein assay kit (G3522, Guangzhou Jiebai Biotechnology Co., Ltd., China). Equal amounts of protein were separated by SDS-PAGE and transferred to PVDF membranes, which were then incubated overnight at 4°C with the following primary antibodies (all from Abcam, UK): *Glucose-fructose oxidoreductase domain-containing protein 2 (GFOD2)* and *E2F transcription factor 2 (E2F2)* (1:500), *Phosphoglucomutase 3 (PGM3)* and *Trifunctional purine biosynthetic protein adenosine-3 (GART)* (1:2000), *ATPase subunit beta-2 (ATP1B2)* (1:1000), *Growth/differentiation factor 15 (GDF15)* (1:10000), and *Transferrin* (1:2000, as loading control). Secondary antibodies were diluted at 1:2000 and incubated at room temperature for 1 h. Immunoreactive bands were visualized using an ECL chemiluminescence detection kit (SB-WB011, Share-Bio, China), and band intensities were quantified using ImageJ software (NIH, USA) to evaluate relative protein expression levels.

### 2.10 Gene set variation analysis

GSVA is a nonparametric and unsupervised algorithm that computes a composite score for gene sets to assess transcriptomic enrichment. This method evaluates functional pathway changes at the gene set level across different samples. The GSVA_1.30.0 package in R was used to calculate t-scores and determine pathway activity states.

### 2.11 Analysis of immune cell abundance

CIBERSORT, based on support vector regression, was used to deconvolute transcriptomic data and estimate the relative abundance of 22 immune cell subtypes defined by the LM22 signature matrix (547 genes). This included various T cells, B cells, plasma cells, and myeloid cells. The algorithm was applied to patient samples to quantify immune infiltration, and correlation analyses were conducted to assess associations between hub gene expression and immune cell composition, thereby exploring potential regulatory roles in the immune microenvironment.

### 2.12 Molecular docking analysis for potential therapeutic drug prediction

Molecular docking was performed to predict potential therapeutic agents targeting the identified hub genes. Candidate compounds were obtained from the DSigDB database via the Enrichr platform (https://maayanlab.cloud/Enrichr/). Three-dimensional structures of target proteins were downloaded from UniProt (https://www.uniprot.org/), and chemical structures of small molecules from PubChem (https://pubchem.ncbi.nlm.nih.gov/).

Docking simulations were carried out using AutoDock (https://ccsb.scripps.edu/mgltools/downloads/) to calculate binding affinities. Each compound was docked 50 times independently, and the top 20 poses from each run were collected. Binding energies were analyzed by calculating the mean, median, standard deviation, interquartile range (IQR), minimum, and maximum to evaluate distribution and reproducibility.Visualization and interaction analysis were performed with PyMOL (https://pymol.org/) and Discovery Studio (BIOVIA, Dassault Systèmes) to identify key ligand–residue interactions, including hydrogen bonds, hydrophobic contacts, and π–π stacking, thereby supporting the reliability of the docking results.

### 2.13 Cell culture and treatments

The human alveolar epithelial cell line A549 (Procell Life Science & Technology Co., Ltd.) was cultured in RPMI-1640 medium supplemented with 10% fetal bovine serum and 1% penicillin–streptomycin. Cells were maintained in a humidified incubator at 37 °C with 5% CO_2_, and the medium was replaced every 2–3 days. When the cells reached the logarithmic growth phase with good viability, they were subjected to subsequent treatments. The cells were divided into four groups: Control, LPS (1 µg/mL for 12 h), LPS+Celastrol (1 µg/mL LPS for 12 h followed by 100 nM Celastrol for 24 h), and LPS+Thiostrepton (1 µg/mL LPS for 12 h followed by 2 µM Thiostrepton for 24 h).

### 2.14 Cell plate clone formation assay

A total of 800 cells per well were seeded into 6-well plates and cultured for 2 weeks at 37°C in a humidified atmosphere containing 5% CO_2_. At the endpoint, the cells were fixed with 4% paraformaldehyde for 20 min and subsequently stained with 0.05% crystal violet (Sigma, USA) for 30 min. The results were evaluated by counting the number of stained viable cells and by measuring the absorbance of the eluted dye at 570 nm.

### 2.15 Cell apoptosis assay

Cell apoptosis was assessed using Annexin V-FITC/PI double staining followed by flow cytometry. Briefly, cells were harvested into centrifuge tubes and washed twice with pre-cooled PBS. The procedure was performed according to the manufacturer’s instructions of the Annexin V-FITC/PI apoptosis detection kit, and apoptotic cells were then analyzed using a flow cytometer.

### 2.16 Statistical analysis

All experiments were performed in triplicate, and data are expressed as the mean ± SEM. Statistical analyses were conducted using GraphPad Prism 10.2.0 (GraphPad Software, Inc.). Comparisons among multiple groups were performed by one-way ANOVA followed by Tukey’s *post hoc* test. A value of p < 0.05 was considered statistically significant.

## 3 Results

### 3.1 DEG screening and WGCNA module analysis

**Figure 2:**
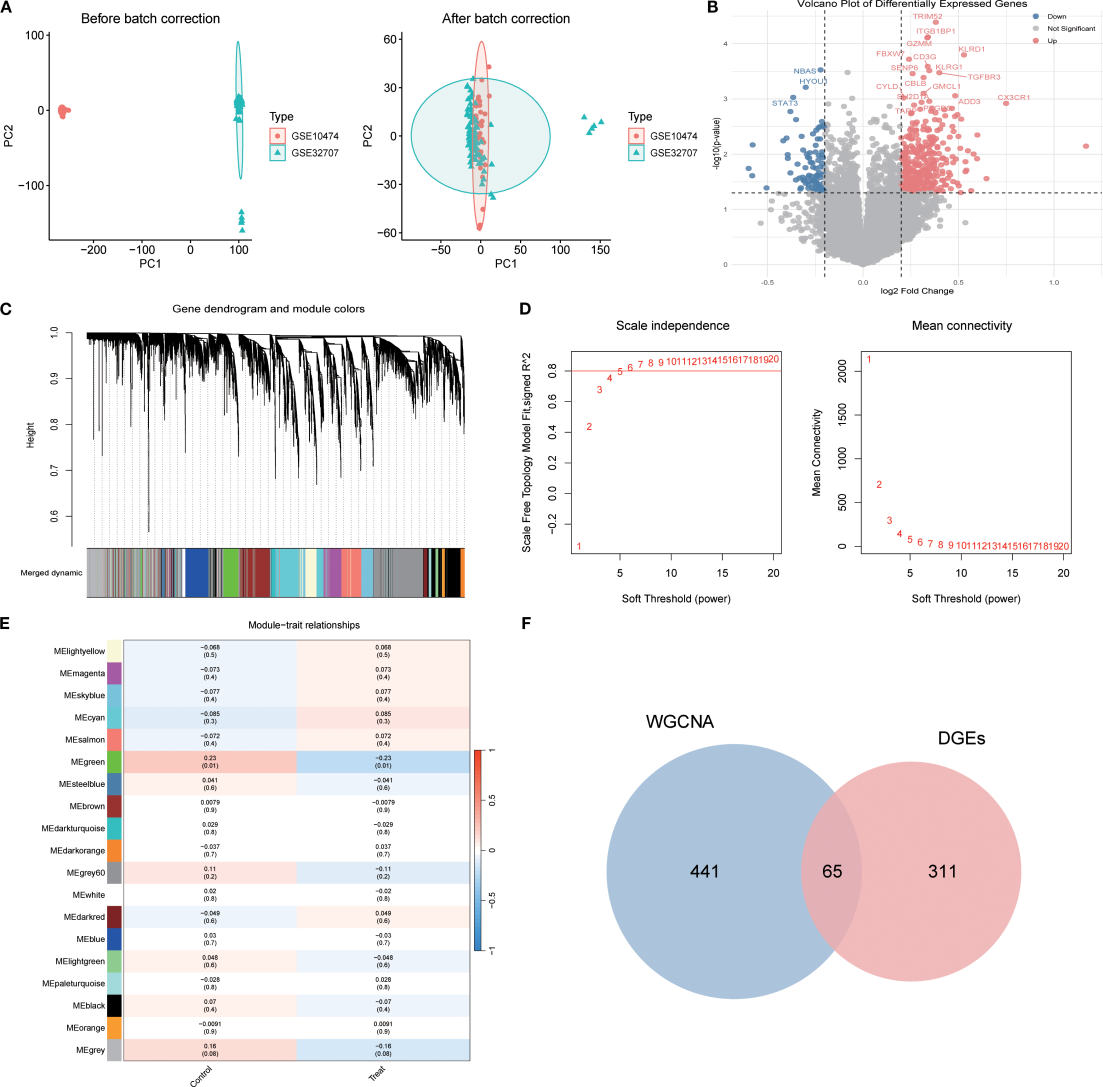
Identification of Differentially Expressed Genes in Sepsis-Associated Acute Lung Injury (SA-ALI). **(A)** PCA of GSE10474 and GSE32707 before (left) and after (right) batch correction; **(B)** Volcano plot of DEGs between SA-ALI and controls (red: upregulated, blue: downregulated, grey: non-significant); **(C)** WGCNA gene clustering dendrogram with module colors; **(D)** Scale-free topology and mean connectivity plots; soft-threshold power = 4 was selected; **(E)** Heatmap of module–trait correlations (Sepsis vs. SA-ALI); **(F)** Venn diagram showing 65 overlapping genes between WGCNA modules and DEGs.

Integrated analysis of the GSE10474 and GSE32707 datasets from the GEO database was performed. PCA revealed pronounced batch effects, which were corrected using the sva package, markedly improving data consistency and reducing inter-sample variability (**Figure 2A**). Differential expression analysis with the limma package, applying thresholds of |log_2_FC| > 0.25 and p < 0.05, identified 376 DEGs, including 166 upregulated and 98 downregulated genes (**Figure 2B**).

WGCNA was then employed to delineate disease-associated gene networks. Sample clustering and the selection of a soft-threshold power of 6 ensured a scale-free topology (**Figures 2C, D**). Multiple color-coded co-expression modules were identified, with the blue module showing a significant positive correlation with disease status (p < 0.01; **Figure 2E**). Intersection of genes from this module with the DEGs yielded 65 overlapping genes, which were designated as core candidates for subsequent functional enrichment and drug–target prediction analyses (**Figure 2F**).

### 3.2 GO and KEGG enrichment analyses of core genes

**Figure 3:**
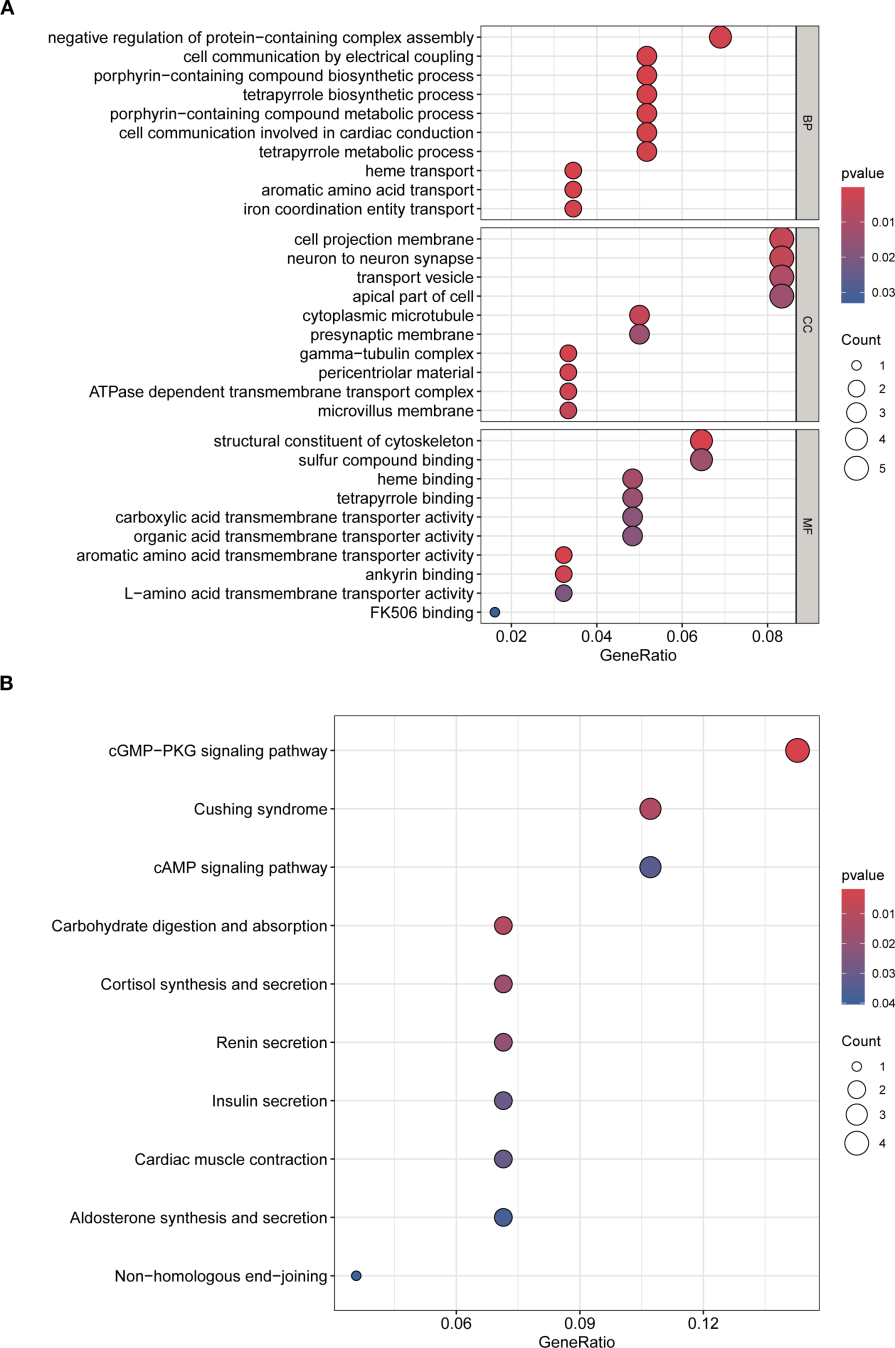
GO and KEGG enrichment analyses of overlapping genes. **(A)** GO analysis of biological processes (BP), cellular components (CC), and molecular functions (MF). **(B)** KEGG pathway enrichment analysis. Dot size indicates gene count, color represents adjusted p-value.

GO enrichment analysis identified key biological processes relevant to SA-ALI, including negative regulation of protein complex assembly, cell–cell electrical coupling, heme transport, and porphyrin-containing compound metabolism, suggesting potential involvement in oxygen metabolism, intercellular signaling, and cardiopulmonary coupling. CC terms such as presynaptic membrane, cytoplasmic microtubule, and ATPase-dependent transmembrane transport complex indicated possible roles in maintaining pulmonary barrier integrity and facilitating intracellular transport. For MF, enrichment in heme binding and structural molecule activity suggested contributions to oxygen delivery and cytoskeletal stability (**Figure 3A**).

KEGG analysis further revealed enrichment in cGMP–PKG and cAMP signaling pathways, cortisol synthesis and secretion, and cardiac muscle contraction, highlighting their potential involvement in vascular tone regulation, stress hormone response, and cardiopulmonary function during SA-ALI. Collectively, these results suggest that the core genes may promote SA-ALI progression by modulating oxygen metabolism, inflammatory signaling, vascular permeability, and cytoskeletal remodeling (**Figure 3B**).

### 3.3 Identification of hub genes and machine learning-based diagnostic biomarkers

**Figure 4:**
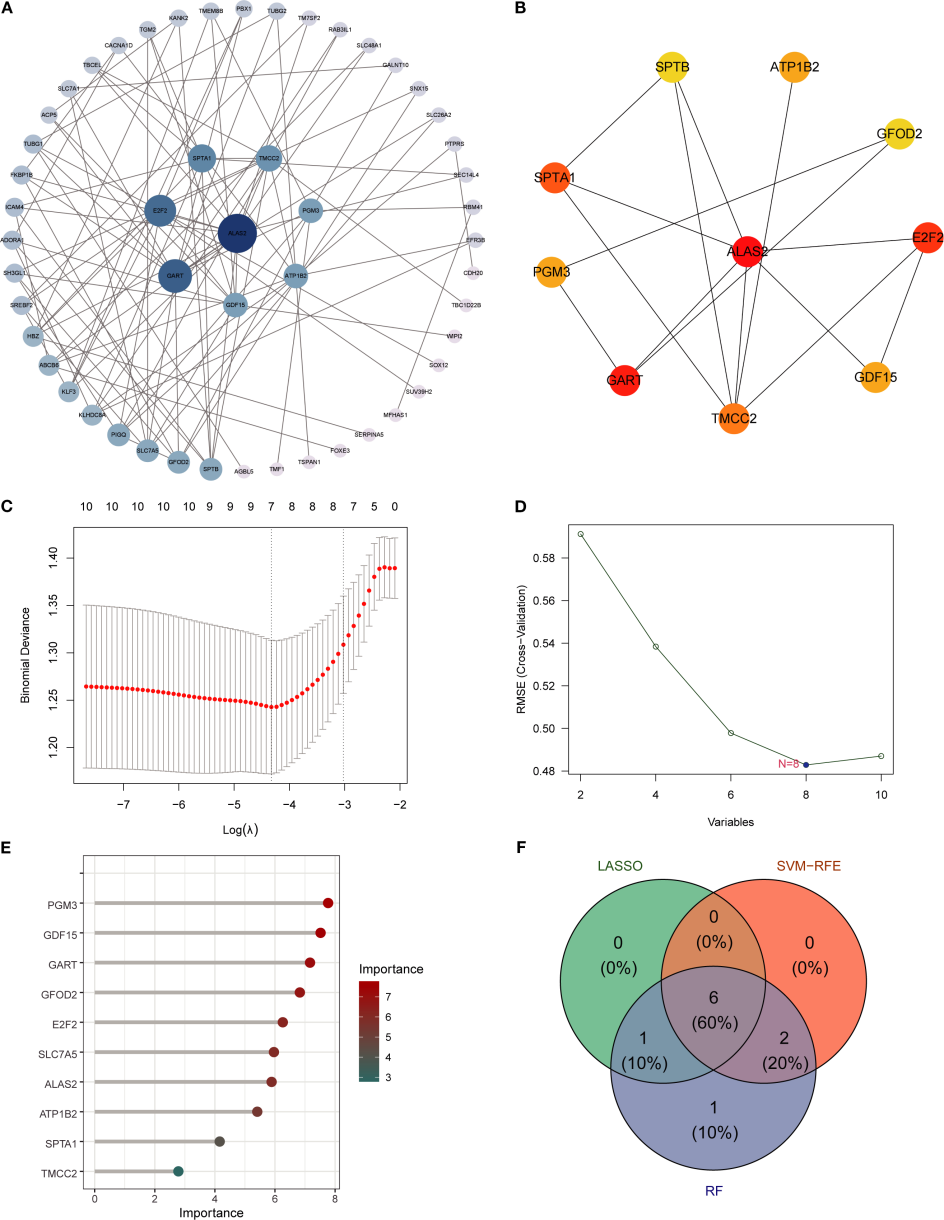
Identification of hub genes using PPI network and machine learning. **(A)** PPI network of overlapping genes, with node size indicating connectivity. **(B)** Top hub genes ranked by cytoHubba MCC algorithm. **(C)** LASSO regression for feature selection. **(D)** SVM-RFE analysis showing minimal RMSE at 8 variables. **(E)** Random Forest ranking of gene importance. **(F)** Venn diagram showing six overlapping hub genes identified by all three algorithms (RF, SVM, LASSO).

A PPI network of SA-ALI–related genes was constructed using the STRING database (minimum interaction score 0.4) and visualized in Cytoscape with CytoHubba, whereby the top 10 hub genes were identified using the Degree algorithm (**Figures 4A, B**). To further screen potential diagnostic markers, three machine learning approaches-LASSO regression, RF, and SVM-RFE-were employed. LASSO regression identified seven genes at the optimal λ of 0.0685, RF ranked the top 10 genes by feature importance, and SVM-RFE selected eight genes with the highest classification accuracy under five-fold cross-validation (**Figures 4C–E**). The intersection of the three methods yielded six candidate biomarkers-*PGM3*, *GDF15*, *GART, GFOD2*, *E2F2*, and *ATP1B2*-which may serve as potential diagnostic and therapeutic targets for SA-ALI (**Figure 4F**).

### 3.4 Validation of candidate diagnostic genes by western blot

**Figure 5:**
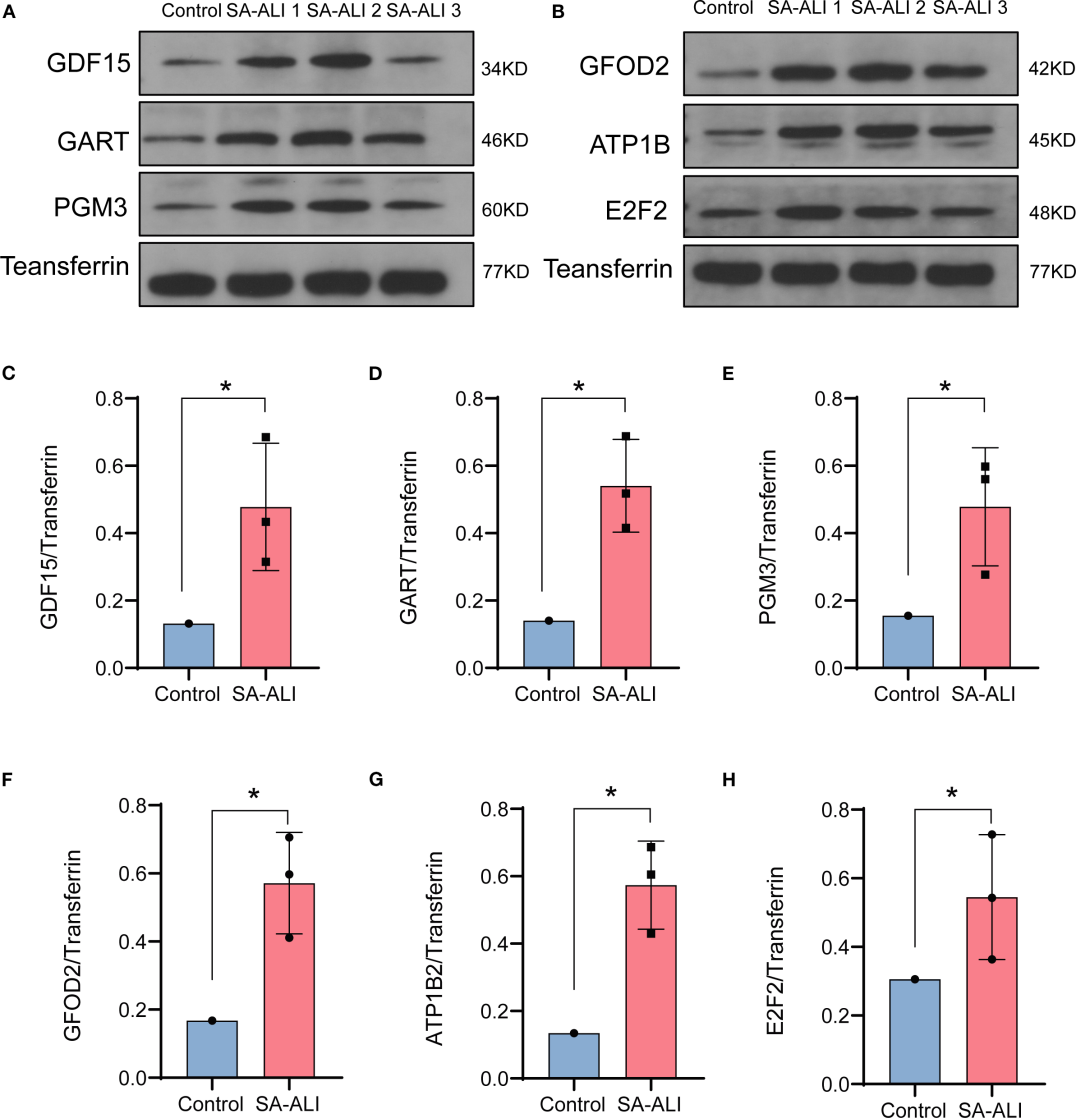
Validation of hub gene expression in SA-ALI by Western blot. **(A, B)** Representative Western blots showing *GDF15*, *GART*, *PGM3*, *GFOD2*, *ATP1B2*, and *E2F2* protein levels in control and SA-ALI patient serum, with transferrin as the loading control. **(C–H)** Quantification of protein expression normalized to transferrin, indicating significant upregulation of all six hub genes in SA-ALI compared with controls (*p < 0.05).

WB analysis was conducted to validate the expression of the six candidate diagnostic genes in clinical serum samples from SA-ALI patients. All six proteins-*GDF15*, *GART*, *PGM3*, *GFOD2*, *ATP1B2*, and *E2F2*-exhibited markedly higher expression levels in SA-ALI patients compared with healthy controls, with Transferrin serving as the loading control (**Figures 5A, B**). Densitometric quantification confirmed significantly elevated expression of each protein in the SA-ALI group (*p* < 0.05) (**Figures 5C–H**), supporting their potential roles as diagnostic biomarkers in SA-ALI.

### 3.5 GSVA reveals pathways associated with key diagnostic genes

**Figure 6:**
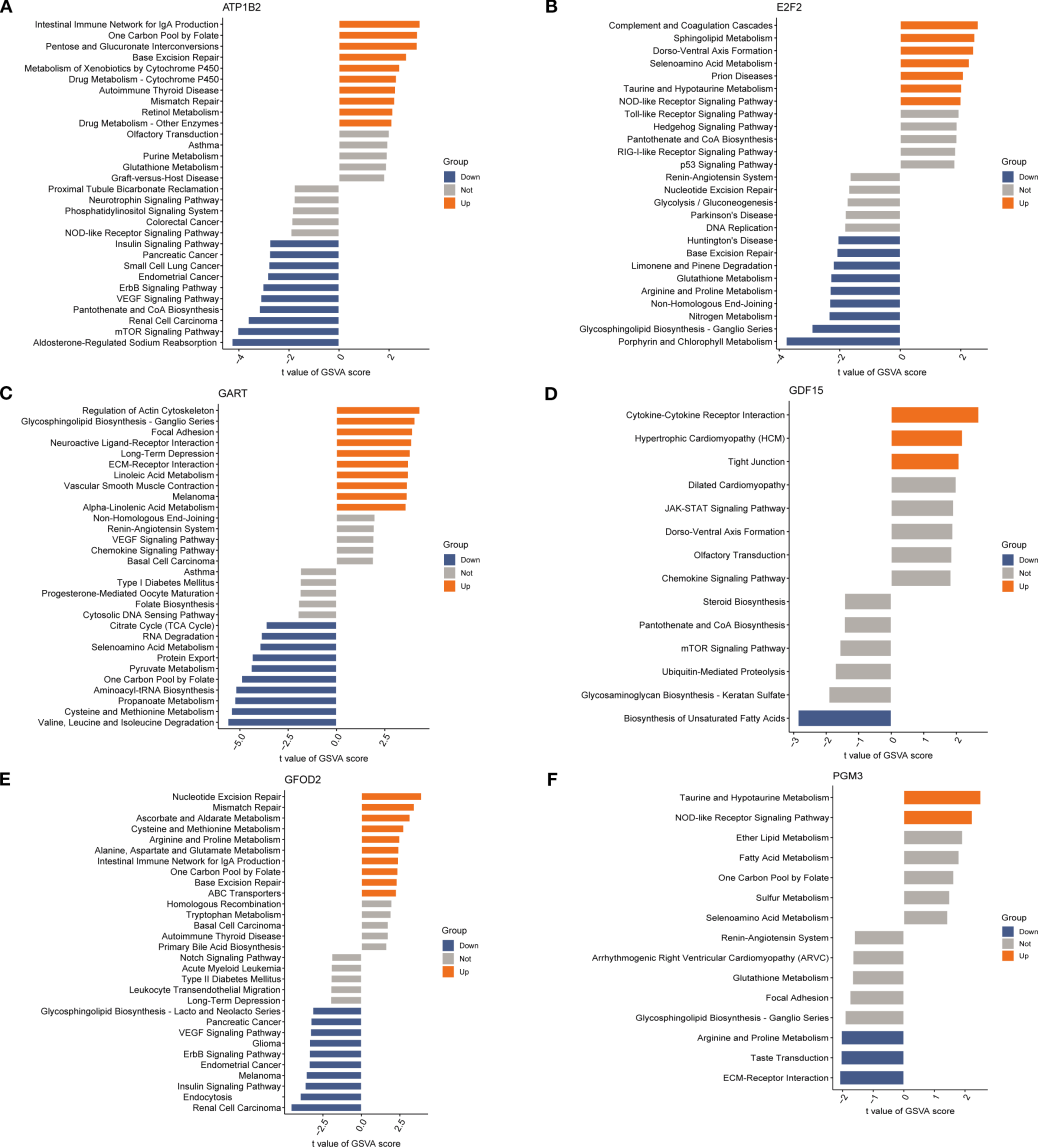
Gene set variation analysis (GSVA) of diagnosis genes: **(A)** GSVA bar chart of *ATP1B2*; **(B)** GSVA bar chart of *E2F2*; **(C)** GSVA bar chart of *GART*; **(D)** GSVA bar chart of *GDF15*; **(E)** GSVA bar chart of *GFOD2*; **(F)** GSVA bar chart of *PGM3*.

To investigate potential functional pathways of the six candidate biomarkers, single-gene pathway enrichment was performed using GSVA (**Figures 6A–F**). *ATP1B2* high expression was associated with intestinal immune network for IgA production, peroxisome, and cytochrome P450 drug metabolism. *E2F2* was enriched in complement and coagulation cascades, Toll-like receptor signaling, and arginine-proline metabolism. *GAR*T correlated with cytoskeletal remodeling, neuroactive ligand–receptor interaction, and ECM-receptor interaction. *GDF15* was linked to cytokine–receptor interaction, tight junction, and JAK-STAT signaling. *GFOD2 was* enriched in nucleotide excision repair, mismatch repair, and cysteine metabolism, whereas *PGM3* was associated with glutathione metabolism, ECM–receptor interaction, and the renin–angiotensin system. These results suggest that the six genes participate in diverse inflammation-, immunity-, metabolism-, and repair-related pathways in SA-ALI.

### 3.6 Immune cell infiltration and association with key genes

**Figure 7:**
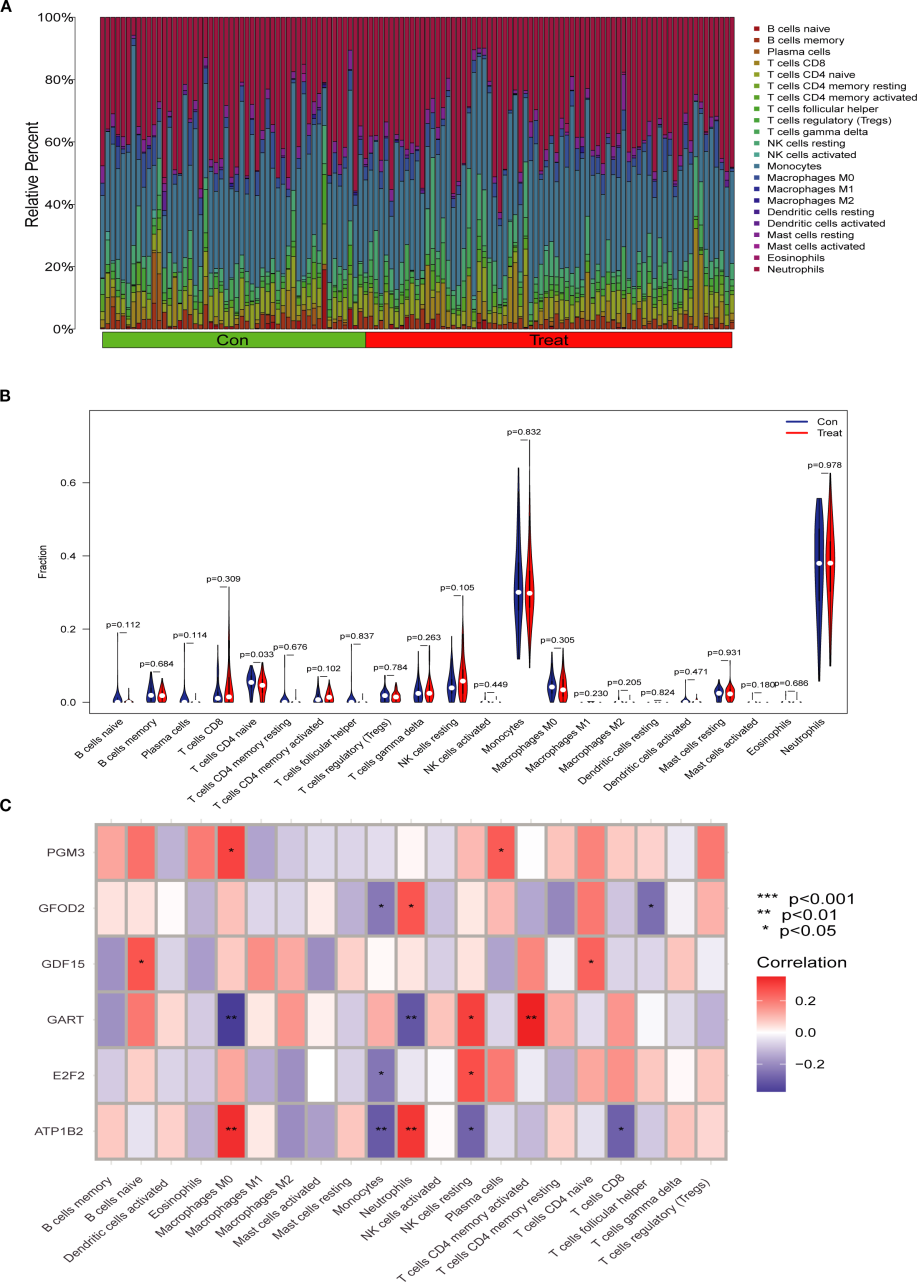
Immune cell infiltration analysis in SA-ALI. **(A)** Stacked bar plot showing the relative proportions of 22 immune cell types in control (Con) and SA-ALI (Treat) samples as estimated by CIBERSORT. **(B)** Violin plots comparing immune cell fractions between groups; significant differences were observed in naïve CD4^+^ T cells (p=0.033). **(C)** Heatmap showing Spearman correlations between hub gene expression and immune cell infiltration levels. Red indicates positive correlation, blue indicates negative correlation; significance levels are marked (*p < 0.05, **p < 0.01, ***p < 0.001).

To explore the immune landscape associated with SA-ALI, we performed immune cell infiltration analysis using the CIBERSORT algorithm. The stacked bar plot revealed substantial heterogeneity in the relative abundance of 22 immune cell types between the control and SA-ALI groups (**Figure 7A**). Violin plot comparison indicated that naive CD4^+^ T cells were significantly reduced in SA-ALI samples compared with controls (p = 0.033), while the proportions of other immune cell types showed no significant differences (**Figure 7B**). Correlation analysis (**Figure 7C**) showed *GDF15* positively associated with naïve CD4^+^ T cells and naïve B cells, while other hub genes displayed distinct links to macrophage subsets, NK cells, and dendritic cells. These findings suggest that hub genes, particularly *GDF15*, may contribute to SA-ALI progression through immune regulation centered on naive CD4^+^ T cell–mediated responses.

### 3.7 Molecular docking analysis of candidate compounds

**Figure 8:**
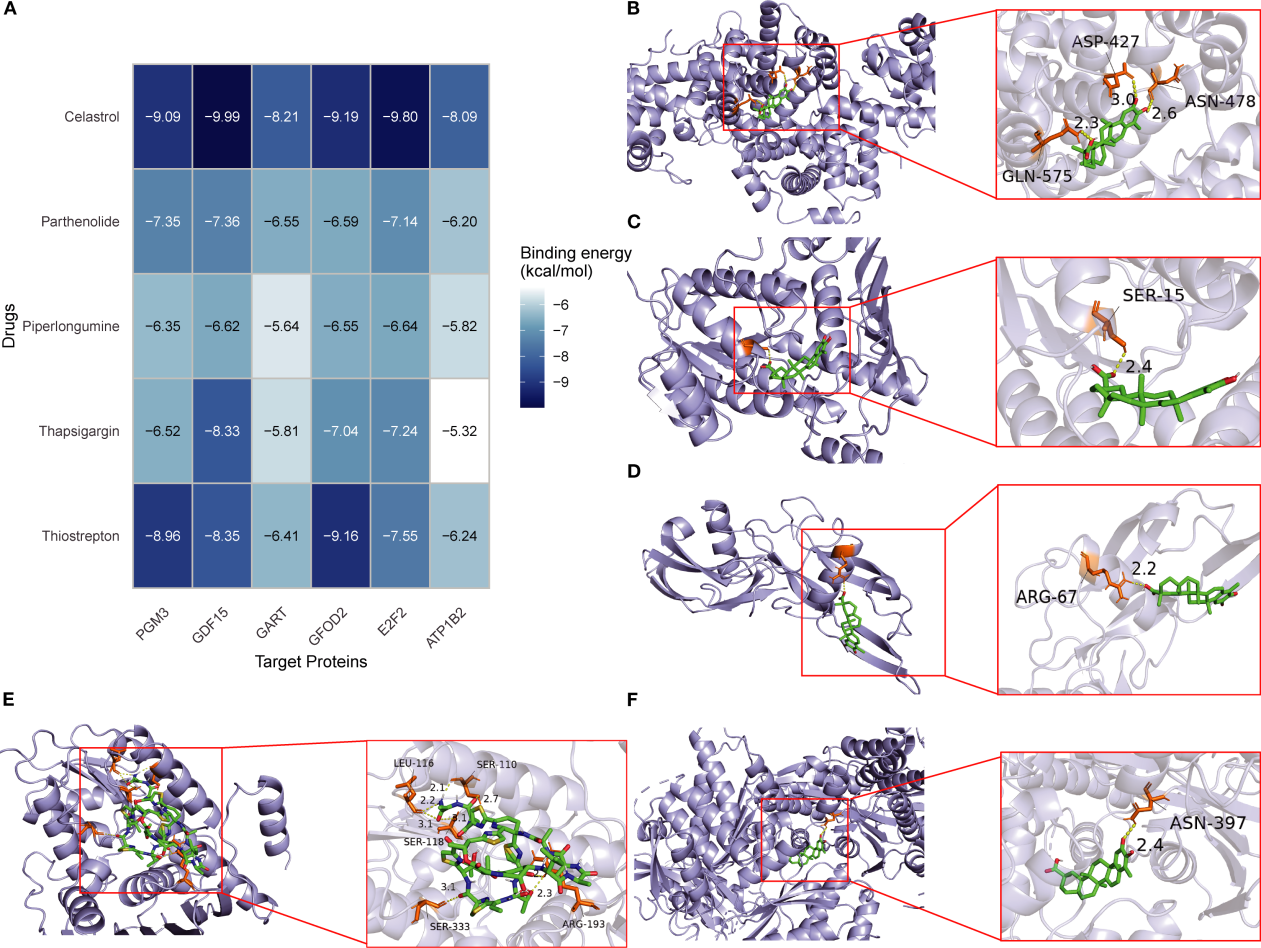
Molecular docking of candidate drugs with hub proteins in SA-ALI. **(A)** Heatmap showing binding energies (kcal/mol) between five candidate compounds (Celastrol, Parthenolide, Piperlongumine, Thapsigargin, and Thiostrepton) and six hub proteins (*PGM3*, *GDF15*, *GART*, *GFOD2*, *E2F2*, *ATP1B2*). Darker blue indicates stronger binding affinity. **(B–F)** Representative docking models of Celastrol with *GDF15* **(B)**, Parthenolide with *PGM3* **(C)**, Piperlongumine with *GART* **(D)**, Thapsigargin with *GFOD2* **(E)**, and Thiostrepton with *E2F2* **(F)**. Green sticks represent ligands; orange sticks represent interacting residues.

Based on DSigDB database screening and literature evaluation, five candidate compounds—thiostrepton, thapsigargin, piperlongumine, parthenolide, and celastrol—were selected for docking with the six core proteins (*PGM3*, *GFOD2*, *GDF15*, *GART*, *E2F2*, and *ATP1B2*) (**Figure 8A**). Binding affinities ranged from -5.0 to -10.0 kcal/mol, with values below -9.0 kcal/mol indicating strong binding (**Figures 8B–F**). Celastrol exhibited high affinity with *PGM3* (-9.086 kcal/mol), *GDF15* (-9.988 kcal/mol), *GFOD2* (-9.185 kcal/mol), and *E2F2* (-9.796 kcal/mol), as well as favorable interactions with *GART* (-8.206 kcal/mol) and *ATP1B2* (-8.093 kcal/mol). Thiostrepton also demonstrated strong binding with *PGM3* (-8.957 kcal/mol), *GFOD2* (-9.156 kcal/mol), and *GDF15* (-8.345 kcal/mol). Based on these results, celastrol and thiostrepton were prioritized for further *in vitro* functional and mechanistic studies.

Statistical analysis of repeated docking runs showed that the binding energy distributions were concentrated, with low standard deviations and narrow interquartile ranges, indicating good stability and reproducibility(Tab S1). Visualization with PyMOL and Discovery Studio further showed that celastrol and thiostrepton formed hydrogen bonds, hydrophobic contacts, and π–π stacking with key amino acid residues, supporting the reliability of the docking results (**Supplementary Figure S1**).

### 3.8 Celastrol and Thiostrepton attenuate LPS-induced apoptosis in A549 alveolar epithelial cells

**Figure 9:**
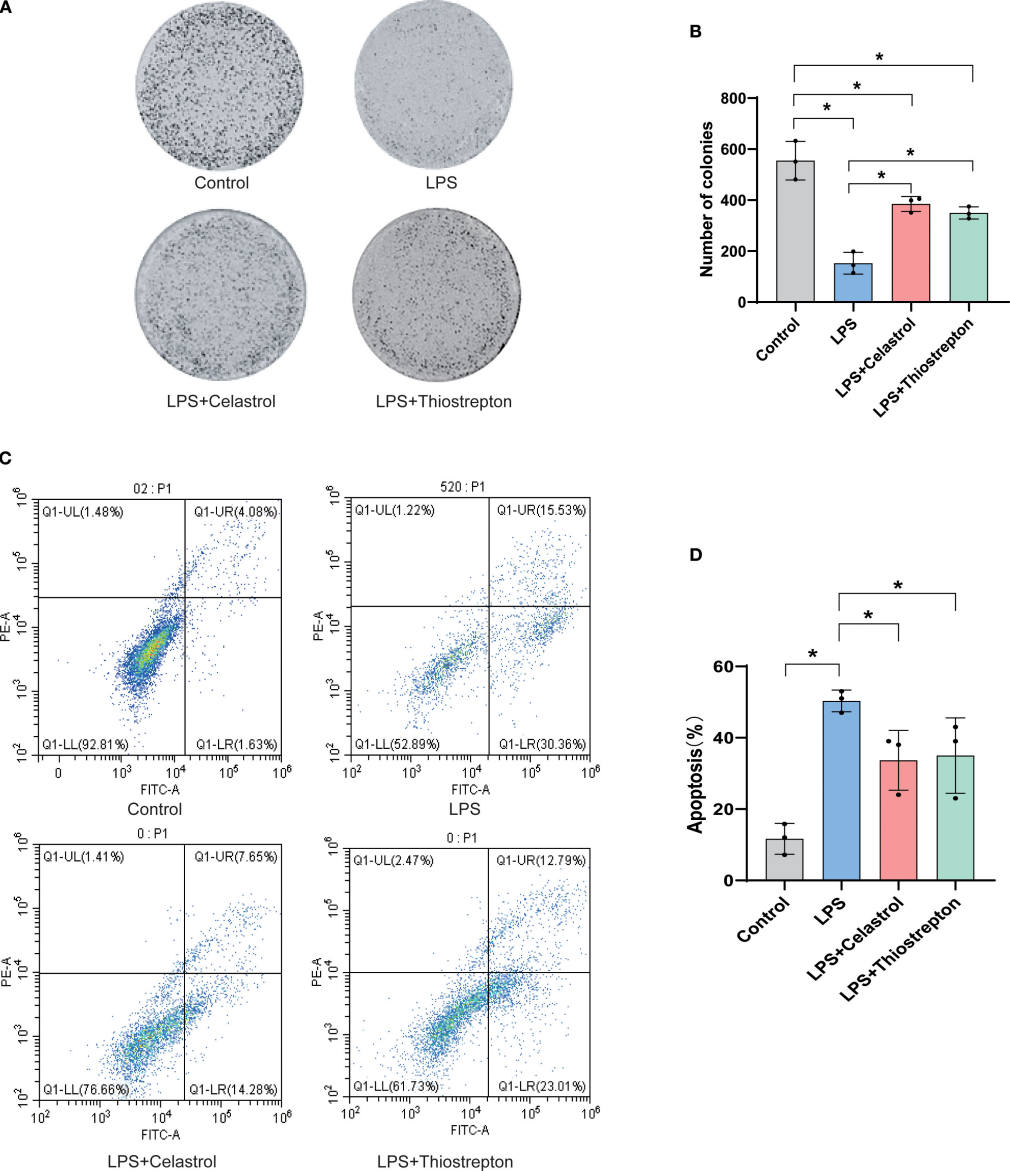
Celastrol and Thiostrepton attenuate LPS-induced apoptosis in alveolar epithelial cells (A549). **(A)** Representative images of cell colony formation assay in Control, LPS, LPS+Celastrol, and LPS+Thiostrepton groups. **(B)** Quantification showing that LPS markedly reduced the clonogenic capacity of alveolar epithelial cells, which was partially restored by Celastrol or Thiostrepton. **(C)** Representative Annexin V-FITC/PI flow cytometry plots displaying early and late apoptotic populations. **(D)** Quantification of apoptosis rates demonstrating that Celastrol and Thiostrepton reduced LPS-induced apoptosis. Data are presented as mean ± SD; *p < 0.05.

Our results showed that LPS stimulation markedly impaired the clonogenic capacity of A549 alveolar epithelial cells, whereas treatment with Celastrol or Thiostrepton partially restored cell growth (**Figures 9A, B**). Flow cytometry analysis further demonstrated that LPS induced a significant increase in both early and late apoptotic populations, while administration of Celastrol or Thiostrepton reduced apoptotic cells and overall apoptosis rates (**Figures 9C, D**). Quantitative analysis confirmed that these differences were statistically significant compared with the LPS group (p < 0.05). Collectively, these findings indicate that Celastrol and Thiostrepton protect A549 alveolar epithelial cells against LPS-induced apoptosis.

## 4 Discussion

During sepsis, the lung is among the most severely affected organs, and SA-ALI can progress to ARDS with high mortality (8, 13, 14). To identify potential biomarkers and therapeutic targets, we analyzed datasets GSE10474 and GSE32707, yielding 376 differentially expressed genes. Integrating WGCNA with three machine learning algorithms, we identified six diagnostic genes—*PGM3*, *GDF15*, *GART*, *GFOD2*, *E2F2*, and *ATP1B2*—associated with sepsis-induced ALI, which were validated in clinical samples by Western blotting. These genes are implicated in immune regulation, inflammation, and cellular stress, with CD4^+^ T cell reduction potentially contributing to lung injury. Drug enrichment and molecular docking further identified Thiostrepton and Celastrol as promising multi-target candidates for SA-ALI intervention. Our findings provide novel biomarkers for early diagnosis and potential agents for targeted therapy.

WB analysis demonstrated that all six key genes were significantly upregulated in SA-ALI patients compared with healthy controls, suggesting their involvement in disease onset and progression. Among them, *GDF15* and *E2F2* have been previously implicated in SA-ALI. *GDF15*, a stress-induced immunomodulatory hormone, is essential for survival during bacterial and viral infections as well as sepsis (15, 16), and may exert protective effects in SA-ALI through multiple mechanisms: (i) activating the AMPK pathway to suppress glycolysis and NF-κB/MAPK signaling, thereby attenuating alveolar macrophage–mediated inflammation; (ii) enhancing its own expression via the eIF2α–ATF4 pathway to establish an anti-inflammatory feedback loop; (iii) inhibiting the HIF-1α/LDHA axis to correct immunometabolic imbalance; and (iv) upregulating SIRT1 to protect alveolar epithelial cells (17–20). Similarly, recent evidence indicates that *E2F2* may also participate in sepsis-related tissue protection by promoting M2 macrophage polarization and suppressing NF-κB signaling (21). In a rat CLP model, activation of the ghrelin/GHSR axis enhanced *E2F2* expression, reduced inflammation, and mitigated intestinal barrier injury, suggesting that *E2F2*-mediated immunomodulation could have broader relevance in SA-ALI (22).

Although no direct evidence currently links *PGM3*, *GART*, *GFOD2*, or *ATP1B2* to SA-ALI, their known biological functions suggest potential involvement in disease pathogenesis. *PGM3*, encoding phosphoglucomutase 3, is a key enzyme in the hexosamine biosynthetic pathway, regulating protein glycosylation, immune cell differentiation, and cytokine production; dysregulation of this pathway can impair immune homeostasis and barrier integrity—critical processes in ALI progression (23, 24). *GART* encodes a trifunctional enzyme in the *de novo* purine biosynthesis pathway, essential for nucleic acid synthesis, cell proliferation, and energy metabolism; enhanced purine metabolism may sustain hyperactivated immune cell proliferation and inflammatory mediator production, exacerbating the septic inflammatory respons (25, 26). *GFOD2*, a glutamate-rich protein with incompletely defined functions, has been implicated in oxidative stress regulation and mitochondrial homeostasis, processes closely related to alveolar epithelial cell survival under inflammatory injury (27). *ATP1B2* encodes the β2 subunit of Na^+^/K^+^-ATPase, a crucial ion pump that maintains transmembrane electrochemical gradients, facilitates alveolar fluid clearance, and preserves epithelial barrier integrity; Na^+^/K^+^-ATPase dysfunction is a recognized mechanism of pulmonary edema in ALI (28, 29). In this study, all four genes were significantly upregulated in SA-ALI patient samples compared with healthy controls, suggesting that they may contribute to disease progression through coordinated effects on immune activation, metabolic reprogramming, oxidative stress regulation, and epithelial barrier function. These findings provide new leads for elucidating their mechanistic roles in SA-ALI and evaluating their potential as therapeutic targets.

Furthermore, we performed immune cell infiltration analysis and found that naïve CD4^+^ T cells play a central role in ALI immunity, differentiating into Th1, Th2, Th17, and Treg subsets that modulate disease progression and resolution (30, 31). In our cohort, *GDF15* expression showed a strong positive correlation with naïve CD4^+^ T cell abundance. Previous studies have demonstrated that *GDF15* promotes FOXP3^+^ induced Treg differentiation, enhances the anti-inflammatory activity of natural Tregs, and suppresses dendritic cell activation, thereby fostering immune tolerance (32). Collectively, these mechanisms may account for the protective role of *GDF15* in SA-ALI through the expansion of immunosuppressive T cell populations and the attenuation of excessive inflammation.

Molecular docking identified two potential therapeutic compounds for SA-ALI—Celastrol and Thiostrepton. Celastrol demonstrated strong binding affinities with all six hub proteins, with the lowest binding energies observed for *PGM3*, *GDF15*, *GFOD2*, and *E2F2*, suggesting multi-target cooperative regulation. As a triterpenoid, Celastrol has been reported to suppress NF-κB/MAPK inflammatory signaling and activate the Nrf2/HO-1 antioxidant pathway in sepsis-induced lung injury, thereby reducing inflammation, inhibiting apoptosis, and improving lung function (33–35). The mechanism of *GDF15*, which activates AMPK to inhibit NF-κB/MAPK signaling in alveolar macrophages, and that of *E2F2*, which amplifies inflammatory responses via the TLR4/MyD88/NF-κB axis, align closely with Celastrol’s anti-inflammatory actions, suggesting potential synergistic effects (17, 18, 22). Furthermore, Celastrol’s ability to activate Nrf2/HO-1 may complement *GFOD2*-mediated oxidative stress regulation, enhancing antioxidant defenses and reducing oxidative damage (35, 36). Although the precise roles of *PGM3*, *GART*, and *ATP1B2* in SA-ALI remain unclear, their favorable binding to Celastrol implies potential indirect benefits through metabolic homeostasis and membrane function regulation.

Thiostrepton, a thiazole-containing macrolide antibiotic, possesses both antimicrobial and immunomodulatory properties. It can inhibit the NF-κB pathway, reduce pro-inflammatory cytokines such as TNF-α and IL-6, and activate FOXO3 and antioxidant pathways to mitigate inflammation and oxidative stress (37–39). Thiostrepton exhibited strong binding with *PGM3*, *GDF15*, and *GFOD2* (binding energies of –8.957, –8.345, and –9.156 kcal/mol, respectively), supporting its multi-target potential. While direct evidence is currently lacking, the strong molecular docking affinities observed between Thiostrepton and *PGM3*, *GDF15*, and *GFOD2* suggest potential mechanistic interactions. It is plausible that Thiostrepton may modulate immune receptor glycosylation via *PGM3*, synergize with *GDF15* to suppress inflammatory signaling, and enhance *GFOD2*-mediated antioxidant defenses to reduce ROS-induced lung injury. However, these hypotheses require experimental validation in future studies. Collectively, these findings highlight Celastrol and Thiostrepton as promising agents with complementary and synergistic anti-inflammatory, antioxidant, and immunoregulatory properties. Their therapeutic potential in SA-ALI warrants further mechanistic investigation and preclinical validation.

Taken together, our identification of six hub genes and their associated pathways not only elucidates the molecular mechanisms underlying SA-ALI but also provides novel opportunities for clinical translation. These biomarkers hold promise for improving the sensitivity and specificity of early diagnosis, enabling clinicians to stratify high-risk patients and initiate timely interventions before progression to ARDS. In addition, the discovery of Celastrol and Thiostrepton as potential multi-target therapeutic agents underscores the feasibility of drug repurposing strategies for SA-ALI. These compounds not only offer mechanistic insights into immunomodulation and oxidative stress regulation but also represent tangible candidates for preclinical and translational evaluation.

Future studies should focus on validating these biomarkers in large, multicenter clinical cohorts, integrating them into predictive models alongside conventional clinical parameters, and assessing their dynamic changes during disease progression. Moreover, systematic preclinical investigations of Celastrol and Thiostrepton—including pharmacokinetic, safety, and efficacy evaluations in animal models, followed by early-phase clinical trials—will be critical to establish their therapeutic potential. By bridging bioinformatics-driven discovery with clinical application, our findings lay the groundwork for precision medicine strategies aimed at improving outcomes in patients with sepsis-associated acute lung injury.

### 4.1 Limitations

Several limitations should be acknowledged. First, the transcriptomic analyses were derived from publicly available datasets with relatively small sample sizes, which may restrict statistical power and generalizability. Second, although the integration of WGCNA, multiple machine learning algorithms, and molecular docking provided a robust strategy for identifying key genes and candidate therapeutics, the findings remain correlative and predictive. Third, our clonogenic and flow cytometric apoptosis assays demonstrated that Celastrol and Thiostrepton exert protective effects on LPS-treated A549 cells; however, their interactions with putative targets were only suggested by molecular docking, and their efficacy and mechanisms require further validation in relevant *in vitro* and *in vivo* SA-ALI models. Fourth, the observed association between GDF15 and naïve CD4^+^ T cells warrants mechanistic studies to establish causality. Finally, the clinical validation was limited to a small cohort, and larger multicenter studies are needed to confirm the diagnostic and therapeutic potential of the identified biomarkers and compounds.

## 5 Conclusion

We identified six key genes—*PGM3*, *GDF15*, *GART*, *GFOD2*, *E2F2*, and *ATP1B2*—closely associated with the pathogenesis of SA-ALI, involving immune regulation, inflammatory signaling, oxidative stress, and epithelial barrier maintenance. Celastrol and Thiostrepton emerged as potential multi-target agents with complementary protective effects. Together, these findings not only provide novel biomarkers for disease monitoring and mechanistic insight into SA-ALI, but also point to promising therapeutic candidates that may inform the development of more effective treatment strategies.

## Data Availability

The datasets analyzed in this study are publicly available in the NCBI Gene Expression Omnibus (GEO) repository under accession numbers GSE10474 and GSE32707 (https://www.ncbi.nlm.nih.gov/geo/query/acc.cgi).
