# Supplementary figures and images for: Integrated bioinformatics and molecular docking analysis reveal potential hub genes and targeted therapeutics in sepsis-associated acute lung injury
Source: Front Immunol. 2025 Oct 10;16:1684774. doi: 10.3389/fimmu.2025.1684774 (PMC12549261; doi:10.3389/fimmu.2025.1684774)

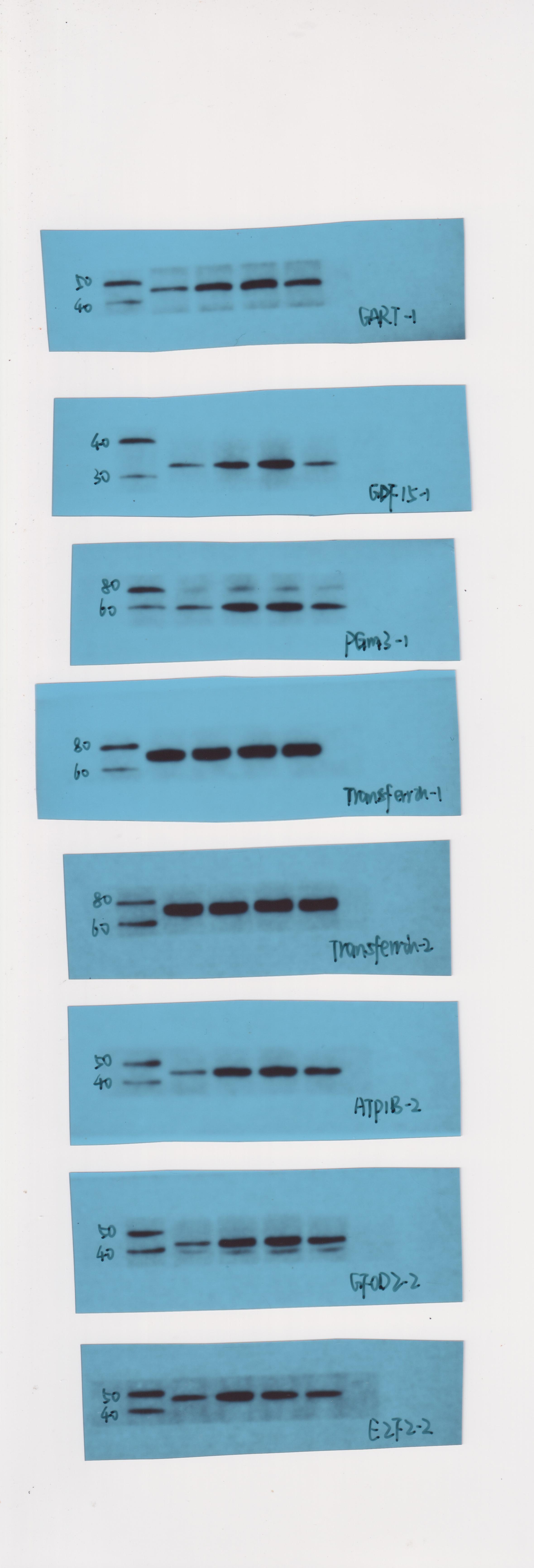

Supplement: Supplementary file 1 [file Image1.jpeg]
