# Supplementary material for: Integrated bioinformatics and molecular docking analysis reveal potential hub genes and targeted therapeutics in sepsis-associated acute lung injury
Source: Front Immunol. 2025 Oct 10;16:1684774. doi: 10.3389/fimmu.2025.1684774 (PMC12549261; doi:10.3389/fimmu.2025.1684774)
Supplement: Supplementary file 2 [file Table1.docx]

1.Cell Plate Clone Formation Assay


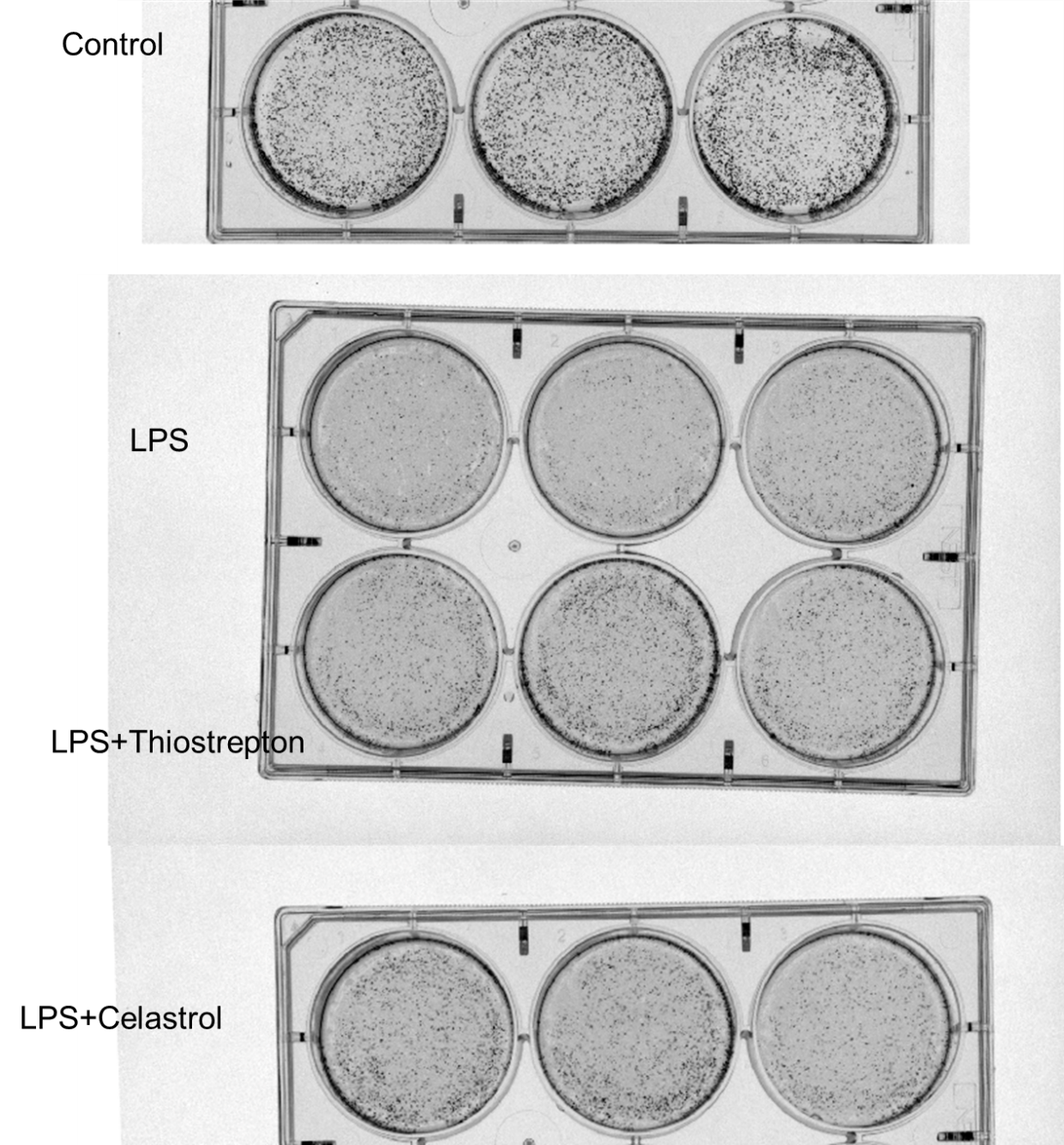


2. Cell apoptosis assay


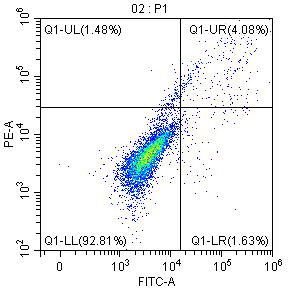

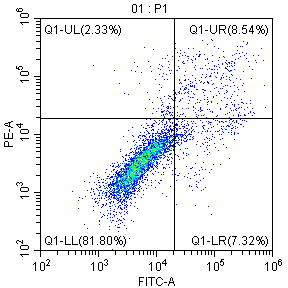

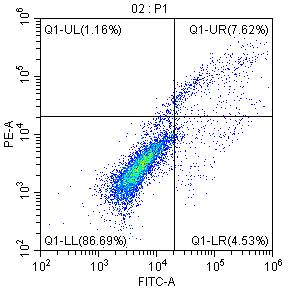


Control


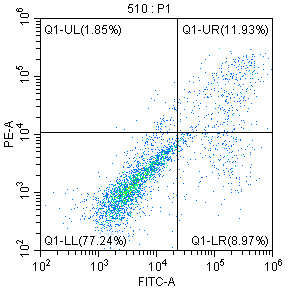

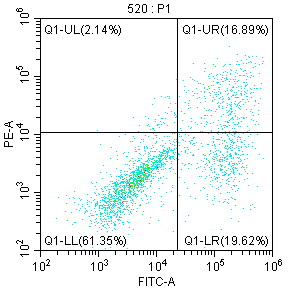

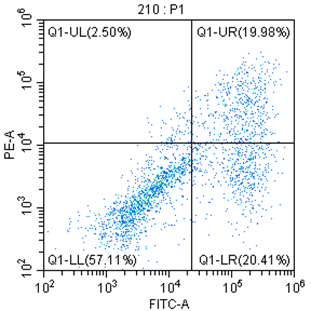


LPS+Thiostrepton


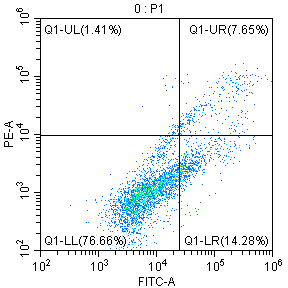

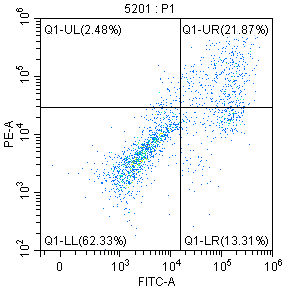

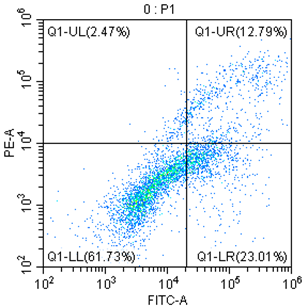


LPS+Celastrol


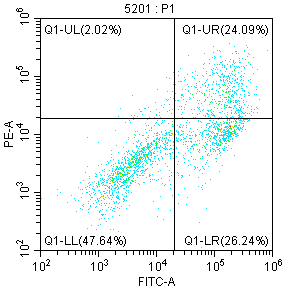

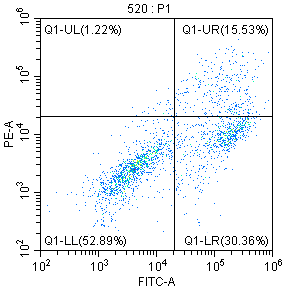

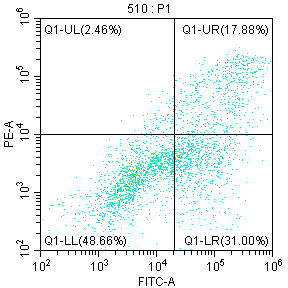


LPS
