# Supplementary material for: Integrated bioinformatics and molecular docking analysis reveal potential hub genes and targeted therapeutics in sepsis-associated acute lung injury
Source: Front Immunol. 2025 Oct 10;16:1684774. doi: 10.3389/fimmu.2025.1684774 (PMC12549261; doi:10.3389/fimmu.2025.1684774)
Supplement: Supplementary file 3 [file Table2.docx]

| Compound | Target Gene | Runs | Top Poses | Box Size (Å³) | Center coordinates (X, Y, Z) | Mean  (kcal/mol) | Median  (kcal/mol) | SD  (kcal/mol) | IQR  (kcal/mol) | Min  (kcal/mol) | Max  (kcal/mol) |
| --- | --- | --- | --- | --- | --- | --- | --- | --- | --- | --- | --- |
| celastrol | PGM3 | 50 | 20 | 62×78×60 | (−0.447, −18.968, 20.618) | −8.068 | −7.916 | 0.425 | 0.592 | −9.086 | −7.474 |
|  | GDF15 | 50 | 20 | 46×50×32 | (31.422, −7.087, −15.487) | −7.229 | −7.101 | 0.546 | 0.852 | −8.206 | −6.522 |
|  | GFOD2 | 50 | 20 | 40×52×52 | (−4.379, −0.291, 0.110) | −8.505 | −8.386 | 0.72 | 1.271 | −9.988 | −7.640 |
|  | E2F2 | 50 | 20 | 54×56×82 | (2.749, 0.834, 20.534) | −8.771 | −8.825 | 0.674 | 1.006 | −9.796 | −7.688 |
|  | GART | 50 | 20 | 102×52×92 | (−2.541, 7.859, −2.135) | −7.709 | −7.589 | 0.588 | 0.822 | −9.185 | −6.882 |
|  | ATP1B2 | 50 | 20 | 54×34×74 | (-5.566, 1.446, -22.582) | −6.687 | −6.467 | 0.568 | 0.785 | −8.093 | −5.915 |
| Thiostrepton | PGM3 | 50 | 20 | 62×78×60 | (−0.447, −18.968, 20.618) | −8.093 | −7.988 | 0.398 | 0.556 | −8.957 | −7.554 |
|  | GDF15 | 50 | 20 | 46×50×32 | (31.422, −7.087, −15.487) | −5.996 | −6.057 | 0.232 | 0.336 | −6.412 | −5.612 |
|  | GFOD2 | 50 | 20 | 40×52×52 | (−4.379, −0.291, 0.110) | −7.429 | −7.353 | 0.507 | 0.454 | −8.345 | −6.646 |
|  | E2F2 | 50 | 20 | 54×56×82 | (2.749, 0.834, 20.534) | −6.711 | −6.723 | 0.449 | 0.641 | −7.550 | −6.026 |
|  | GART | 50 | 20 | 102×52×92 | (−2.541, 7.859, −2.135) | −7.704 | −7.501 | 0.544 | 0.384 | −9.156 | −7.238 |
|  | ATP1B2 | 50 | 20 | 54×34×74 | (-5.566, 1.446, -22.582) | −5.369 | −5.232 | 0.482 | 0.696 | −6.236 | −4.697 |

Table S1. Statistical summary of molecular docking results for Celastrol and Thiostrepton with six core target genes.


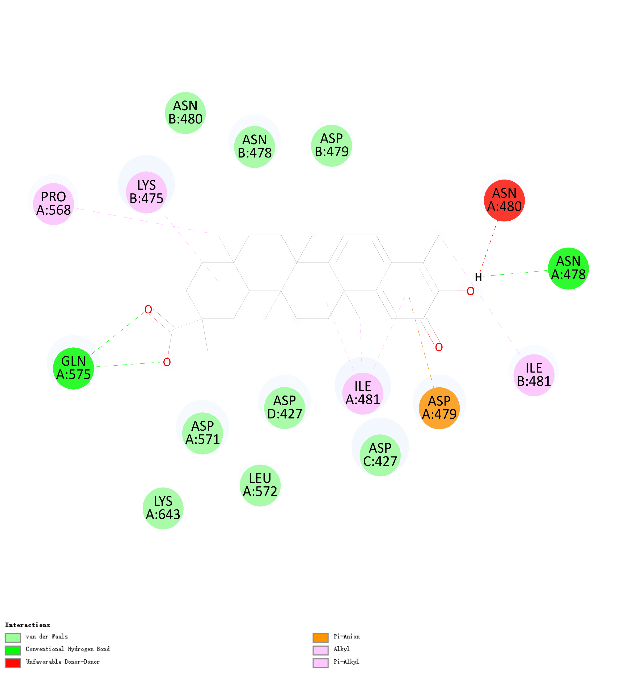


Celastrol-E2F2


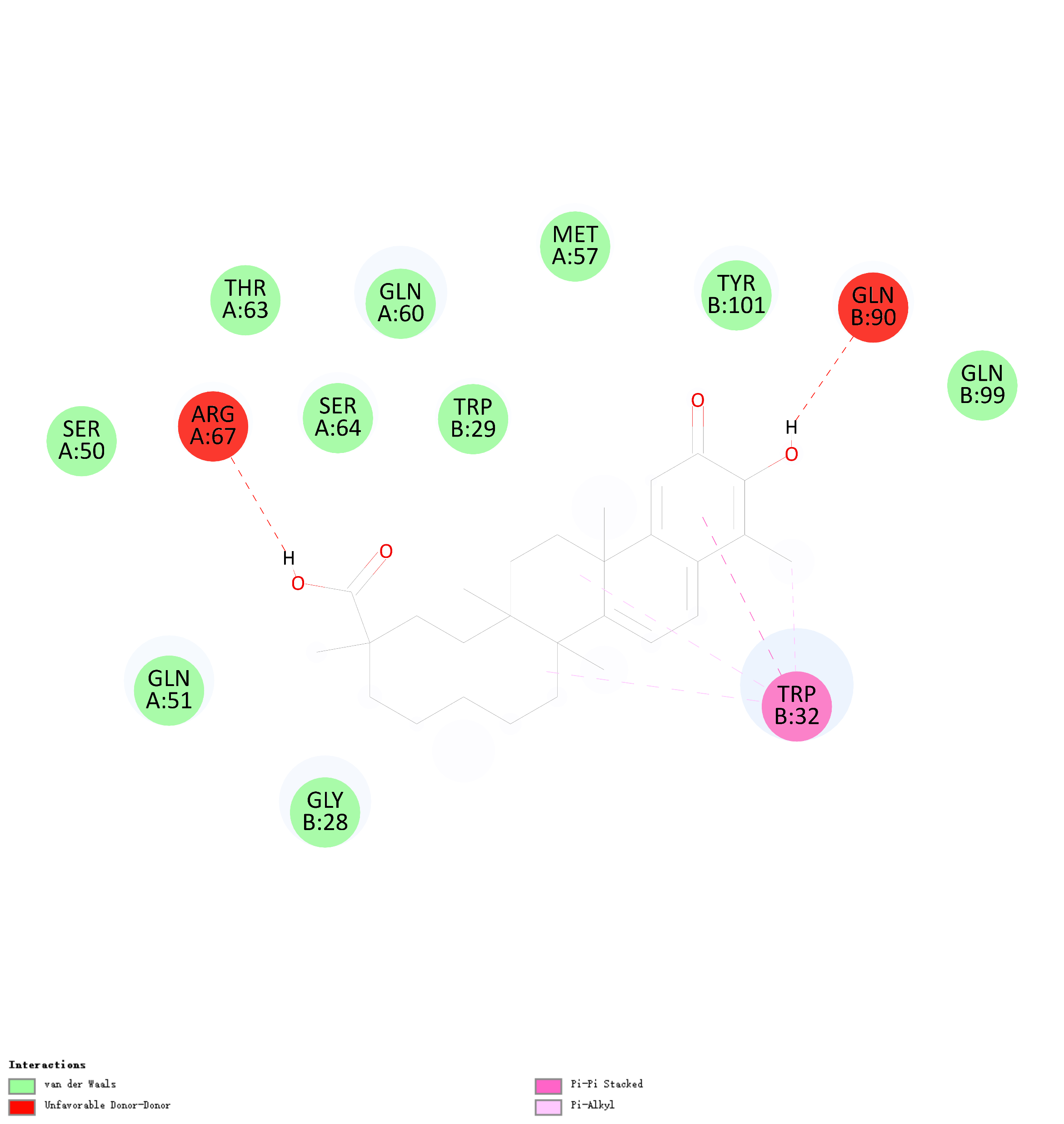


Celastrol-GDF15


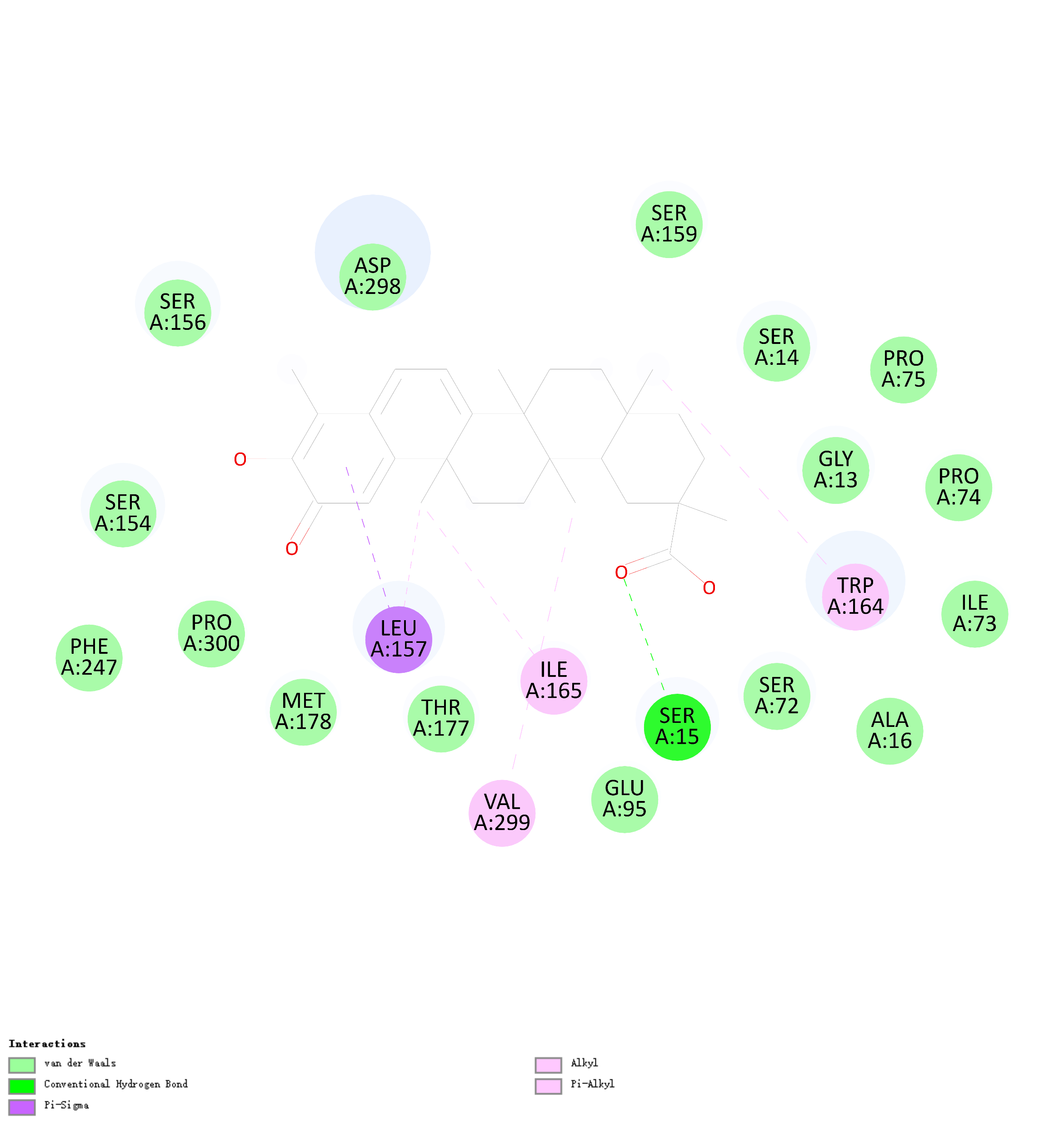


Celastrol-GFOD2


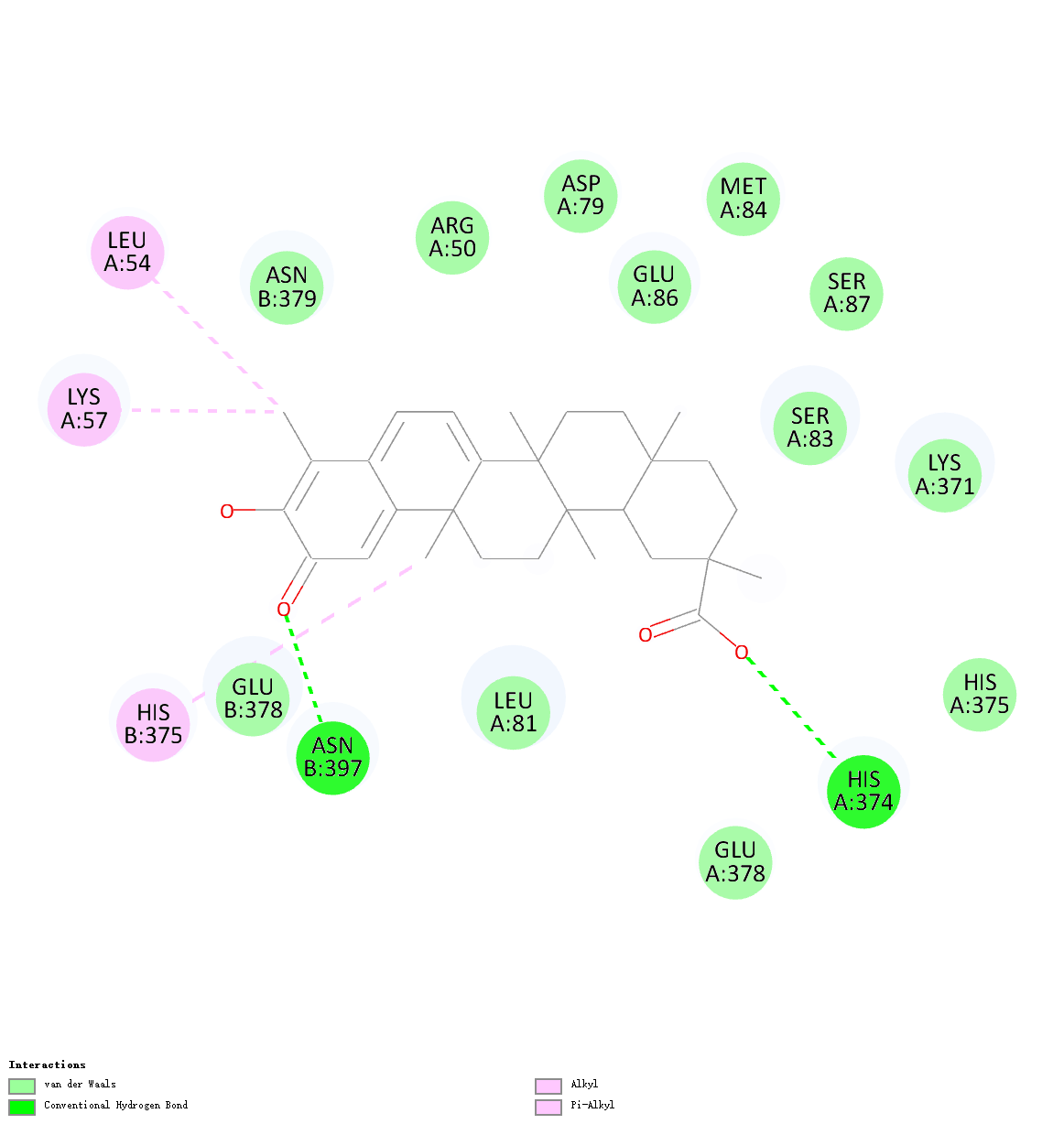


Celastrol-PGM3

Fig S1. Predicted 2D interaction patterns between Celastrol and hub proteins (E2F2, GDF15, and PGM3), illustrating hydrogen bonds, hydrophobic contacts, and π–π stacking interactions with key amino acid residues.
